# Associations between developmental timing of child abuse and conduct problem trajectories in a UK birth cohort

**DOI:** 10.1186/s12888-021-03083-8

**Published:** 2021-03-16

**Authors:** Andreas Bauer, Gemma Hammerton, Abigail Fraser, Graeme Fairchild, Sarah L. Halligan

**Affiliations:** 1 Department of Psychology, 10 West, University of Bath, Claverton Down, Bath, BA2 7AY UK; 2 Population Health Sciences, Bristol Medical School, University of Bristol, Bristol, UK; 3 MRC Integrative Epidemiology Unit, University of Bristol, Bristol, UK

**Keywords:** Child abuse, Child maltreatment, Antisocial behavior, Conduct problems, Developmental trajectories, Latent class growth analysis, ALSPAC

## Abstract

**Background:**

Although there is strong evidence for a relationship between child abuse and neglect and conduct problems, associations between child abuse experienced at different developmental stages and developmental trajectories of conduct problems have not been examined. We sought to investigate effects of timing of child abuse on conduct problem trajectories in a large UK birth cohort study.

**Methods:**

We applied latent class growth analysis to identify conduct problem trajectories in the Avon Longitudinal Study of Parents and Children, using parent-rated conduct problems from ages 4–17 years (*N* = 10,648). Childhood-only and adolescence-only abuse, in addition to abuse in both developmental periods (‘persistent’ abuse), were assessed by retrospective self-report at age 22 years (*N* = 3172).

**Results:**

We identified four developmental trajectories: early-onset persistent (4.8%), adolescence-onset (4.5%), childhood-limited (15.4%), and low (75.3%) conduct problems. Childhood-only abuse and ‘persistent’ abuse were associated with increased odds of being on the early-onset persistent and adolescence-onset conduct problem trajectories compared to the low conduct problems trajectory. Adolescence-only abuse was not predictive of trajectory membership. There were no associations between abuse and childhood-limited trajectory membership.

**Conclusions:**

Early-onset persistent and adolescence-onset conduct problems showed similar patterns of association with abuse exposure, challenging developmental theories that propose qualitative, as opposed to quantitative, differences in environmental risk factors between these trajectories. The results also highlight that childhood-only and ‘persistent’ abuse were more strongly linked to elevated conduct problem trajectories than adolescence-only abuse, and that ‘persistent’ abuse is particularly detrimental.

**Supplementary Information:**

The online version contains supplementary material available at 10.1186/s12888-021-03083-8.

## Background

Conduct problems refer to antisocial behaviors displayed in childhood and/or adolescence that are symptomatic of conduct disorder and oppositional defiant disorder [1]. They account for a substantial proportion of personal [2], familial [3], and societal burden [4–6], and are associated with negative outcomes across multiple domains, including mental and physical health problems [7, 8]. It is therefore crucial to thoroughly understand the etiology of such difficulties and to develop effective prevention and intervention programs.

According to Moffitt’s developmental taxonomic theory [9, 10], individuals with elevated conduct problems can be classified into two subtypes: *early-onset persistent* (also called *‘life-course persistent’*) and *adolescence-limited*. Early-onset persistent conduct problems are proposed to emerge in childhood, originating from genetic, congenital, or acquired neuropsychological deficits. Accumulating interactions with high-risk environments culminate in antisocial and aggressive behavior that persists throughout the lifespan. Thus, neurocognitive impairments, difficult child temperament, and adverse family environments have been proposed as the main risk factors for early-onset persistent conduct problems [10]. By contrast, adolescence-limited conduct problems are considered to be caused by an extended period of dependence, labeled the *maturity gap*, in which the individual is treated as a child despite being biologically mature [9, 10]. This leads them to imitate the behavior of their peers showing early-onset persistent conduct problems. Thus, delinquent peer relationships are proposed to be the main determinant of adolescence-limited conduct problems [10]. Accordingly, while early-onset persistent conduct problems are considered a neurodevelopmental disorder, adolescence-limited conduct problems are viewed as normative and transient – an exaggerated form of normal teenage rebellion [9, 10].

The developmental taxonomic theory has been crucial in shifting focus from considering adolescent conduct problems as a unitary phenomenon to understanding different trajectories of conduct problems that may result from distinct risk factors. Nonetheless, accumulating empirical evidence from a range of disciplines suggests three potential revisions to this model: (i) the addition of a second *adolescence-onset* subtype that emerges in adolescence but persists into adulthood; (ii) the inclusion of a second childhood-onset subtype, in which antisocial behavior remits in the transition from childhood to adolescence (*childhood-limited*); and (iii) the reformulation of the assumption of distinct etiological causes to a model of common individual and environmental risks across subtypes, albeit with different timings and magnitudes of exposure [11]. In sum, whereas the original developmental taxonomic theory proposes a *qualitative* distinction between early-onset persistent and adolescence-limited conduct problems in terms of etiology and developmental course, there is accumulating evidence for additional conduct problem trajectories, including adolescence-onset and childhood-limited, and *quantitative* differences across all subtypes – with children with early-onset persistent conduct problems being exposed to the highest levels of individual and environmental risk and those with adolescence-limited conduct problems exposed to the lowest. More precisely, the magnitude, number, and range of risk factors may be more influential in differentiating between early-onset persistent and adolescence-limited conduct problems than any individual risk factor [12–14].

A key environmental risk factor implicated in the development of conduct problems is child abuse (i.e., physical, psychological, or sexual) and neglect (i.e., physical or psychological), with evidence from prospective longitudinal studies showing that those exposed to abuse and neglect in childhood and/or adolescence are at increased risk of developing conduct problems compared to those who are not exposed [15, 16]. With respect to conduct problem trajectories, several studies have reported that child abuse and/or neglect are associated with childhood-onset conduct problems (i.e., early-onset persistent or childhood-limited), but not those that develop in adolescence (i.e., adolescence-limited or adolescence-onset), which is broadly consistent with the hypothesis of distinct risk factors across these groups [17–19]. By contrast, Odgers et al. [20] reported higher rates of child abuse and neglect in individuals with early-onset persistent, childhood-limited, *and* adolescence-onset conduct problems. Collectively, these studies all provide evidence that child abuse and neglect is associated with the early-onset persistent conduct problems trajectory, but the findings for conduct problems emerging in adolescence are less conclusive. Furthermore, child abuse and neglect was exclusively measured in childhood in these studies, rather than in adolescence or both developmental periods; consequently, existing evidence is limited in terms of understanding the relationship between developmental timing of abuse and different trajectories of conduct problems, especially considering that exposure to abuse may be more common in adolescence than in childhood [21].

Determining how the developmental timing of abuse or its persistence maps onto trajectories of conduct problems may provide new insights into the mechanisms underlying risk for conduct problems. For example, while some have proposed that childhood may be a period of particular sensitivity to adverse rearing environments, due to their potential impacts on neural, cognitive, and social development [22], others have argued that adolescence may be a sensitive period, as it is a key stage of maturation of specific brain regions, such as the medial prefrontal cortex [23, 24]. Alternatively, the *accumulation* of negative experiences may be most relevant in determining outcomes, irrespective of their timing [25]. However, evidence relating to timing or persistence of exposure to abuse in relation to conduct problems is limited to studies examining outcomes in adolescence and adulthood only, and these have yielded mixed findings. Thornberry and colleagues found adolescence-only and persistent abuse to be consistently predictive of adolescent and adult antisocial behavior, whereas childhood-only abuse showed weaker or null associations [26–28]. By contrast, Mersky et al. [29] found childhood-only, adolescence-only, and persistent abuse to be equally predictive of antisocial behavior in adolescents. However, these studies did not investigate conduct problem *trajectories*, meaning that our understanding of the impact of timing of abuse on the longitudinal development and course of conduct problems remains limited.

To address these gaps in the literature, we examined developmental trajectories of conduct problems in a large population-based sample and differentiated between childhood-only and adolescence-only abuse, in addition to abuse occurring in both developmental periods (hereafter referred to as ‘persistent’ abuse). The main objectives of the current study were: (i) to estimate developmental trajectories of conduct problems from ages 4–17 years in a longitudinal population-based sample, extending existing trajectories from the same sample which only covered the period from 4 to 13 years [30]; and (ii) to examine associations between exposure to abuse across childhood and/or adolescence and our derived conduct problem trajectories. We expected *temporal ordering effects*; while exposure to abuse in childhood may predict the subsequent development of conduct problems in adolescence, the converse relationship would not apply. According to this logic, exposure to adolescence-only abuse would be associated with adolescence-onset, but not childhood-limited, conduct problems. Consistent with a *dose-response* or *accumulative effect*, we further hypothesized that ‘persistent’, as opposed to time-limited, abuse would yield the strongest effects for all elevated conduct problem trajectories, especially for the early-onset persistent trajectory, as it may cause the emergence of conduct problems in childhood and contribute to their maintenance in adolescence. For our primary analyses, we used an aggregate measure of abuse, encompassing physical, psychological, and sexual abuse, as the base rates of individual abuse subtypes for some conduct problem trajectories were low in our sample. However, in a set of exploratory analyses, we also investigated whether particular abuse subtypes were more strongly associated with the elevated conduct problem trajectories than others.

## Methods

### Participants

The Avon Longitudinal Study of Parents and Children (ALSPAC) is a prospective birth cohort study, investigating genetic and environmental influences on health and development across the lifespan. All pregnant women residing in the Avon catchment area in South-West England, with an estimated delivery date between April 1991 and December 1992, were eligible for inclusion. Individuals were recruited through media information, community outreach, and promotional material supplied at routine antenatal and maternity health services. Out of 20,248 eligible pregnancies, 14,541 (71.8%) were initially recruited. Of those, 68 had no known birth outcome. The remaining 14,472 pregnancies consisted of 14,676 fetuses, with 14,062 live births, of whom 13,988 were alive at age 12 months. The current sample was restricted to singletons or first-born twins, resulting in an overall sample size of 13,793 participants (51.6% boys). Prior to 2014, questionnaires were sent out to parents/carers by post. If a response was not received within 7 days, two reminder letters were sent and eventually participants were called or visited at their homes. Questionnaires from 2014 onwards were available online or in paper format, and collected and managed using REDCap electronic data capture tools hosted at the University of Bristol [31]. Participants were sent four reminders at three-week intervals. Further details on the cohort can be found elsewhere [32, 33].

### Measures: conduct problems

Conduct problems were measured at ages 4, 7, 8, 10, 12, 13, and 17 years, using the parent-rated conduct problems subscale of the Strengths and Difficulties Questionnaire (SDQ) [34, 35]. This widely used scale consists of five items asking about the child’s behavior over the last six months: (1) “often has temper tantrums or hot tempers”; (2) “generally obedient, usually does what adults request” (reverse coded); (3) “often fights with other children or bullies them”; (4) “often lies or cheats”; and (5) “steals from home, school or elsewhere”. All items are rated on a 3-point scale (0–2), from *not true* to *somewhat true* and c*ertainly true*, yielding overall scores ranging from 0 to 10. Previously reported developmental trajectories of conduct problems from ages 4–13 years in ALSPAC dichotomized the conduct problems subscale as ‘high risk’ versus ‘not high risk’ [30]. In order to maximize variability in conduct problems, but also account for the highly skewed distribution, we used the updated 4-band categorization that has been validated for ages 4–17 years [36], with scores of 0–2 classified as ‘close to average’, 3 as ‘slightly raised’, 4–5 as ‘high’, and 6–10 as ‘very high’. The mean internal consistency was modest (*α* = 0.54, range = 0.50–0.59), which may be attributed in part to the scale’s efforts to cover a wide range of problem behaviors across childhood and adolescence. Nonetheless, in their review, Stone et al. [37] reported a similar value of *α* = 0.58, and demonstrated acceptable reliability and validity of the SDQ conduct problems subscale on the basis of a more rigorous psychometric assessment.

#### Validation of derived conduct problem trajectories

We used the Edinburgh Study of Youth Transitions and Crime (ESYTC) questionnaire to validate the derived conduct problem trajectories [38]. The ESYTC was administered via self-report at ages 14 (*N* = 5604) and 18 (*N* = 3743) years, and included six items, asking, for example, whether the participant “deliberately damaged or destroyed property” or had “broken into a car or van with intention of stealing something out of it”. Items are rated on a 4-point scale, from *not at all*, to *just once*, *2–5 times*, and *6 or more times*. Cronbach’s alphas were 0.52 and 0.45 at ages 14 and 18 years, respectively. We chose to dichotomize this measure – antisocial behavior was either considered ‘present’ (at least *just once* for one or more items) or ‘absent’ (*not at all* for all items) – due to a highly skewed distribution.

### Measures: child abuse

We measured physical, psychological, and sexual abuse occurring in childhood (defined as before age 11 years) and adolescence (defined as between ages 11–17 years) at age 22 years by retrospective self-report. The measure has been used previously in the Growing Up Today Study, a US population-based cohort [39]. Since we were interested in *time-dependent* associations between child abuse and conduct problem trajectories, continuous scales had to be converted into binary variables. Similar to prior research examining the developmental timing of abuse in relation to conduct problems, which distinguished between abuse occurring up to age 11 years and between ages 12–17 years [26–29], we created three abuse exposure categories. These included *childhood-only* (i.e., only before the age of 11 years), *adolescence-only* (i.e., only between ages 11–17 years), and *‘persistent’* abuse (i.e., abuse in both developmental periods). For our primary analysis, we computed an aggregate measure of *any abuse* (i.e., either physical, psychological, or sexual abuse) as preliminary analyses indicated high correlations between abuse subtypes (see Supplementary Fig. 1 for the correlation matrix), in addition to low frequencies of some abuse subtypes. Nonetheless, we also performed exploratory analyses testing for associations between abuse subtypes and conduct problem trajectories to examine whether certain subtypes were more influential than others.

#### Physical abuse

We used two items to assess physical abuse, asking whether an adult in the family “hit you so hard it left you with bruises or marks?” or “actually kicked, punched, or hit you with something that could hurt you, or physically attacked you in another way?”. Items were rated on a 5-point scale from *never* to *rarely*, *sometimes*, *often*, and *very often*. In line with previous studies [40, 41], physical abuse was coded as ‘present’ or ‘absent’.

#### Psychological abuse

Four items were used to assess psychological abuse, asking participants whether an adult in the family “shouted at you?”; “said hurtful or insulting things to you?”; “punished you in a way that seemed cruel?”; and “threatened to kick, punch, or hit you with something that could hurt you or physically attack you in another way?”. Again, items were rated on a 5-point scale (0–4), from *never* to *very often*. Considering the complex nature of psychological abuse, we followed Roberts et al. [41] and computed a sum score ranging from 0 to 16, with participants scoring in the top decile (i.e., scores of ≥7 in our sample) being classified as having experienced psychological abuse.

#### Sexual abuse

We used two items to assess sexual abuse, including “Were you touched in a sexual way by an adult or an older child or were you forced to touch an adult or older child in a sexual way when you did not want to?” and “Did an adult or an older child force you or attempt to force you into any sexual activity by threatening you or holding you down or hurting you in some way when you did not want to?”. In line with previous work [40], sexual abuse was coded as ‘present’ or ‘absent’.

### Covariates

Information on all covariates was collected by maternal self-report during pregnancy, except for child sex, which was obtained from the birth certificate. Housing tenure was assessed at 8 weeks gestation. Participants were asked whether their house was *bought/mortgaged*, *owned*, *rented*, or *other*. We dichotomized this variable into ‘mortgaged/owned’ or ‘other’. Maternal severe depression was assessed at 12 weeks gestation. Participants were asked whether they had ever had severe depression. *Yes, had it recently* and *Yes, in the past, not now* was coded as ‘yes’ and *No, never* was coded as ‘no’. At 18 weeks gestation, mothers were asked whether they had smoked tobacco in the first 3 m of pregnancy. *Cigarettes*, *Cigars*, *Pipe*, and *Other* were coded as ‘yes’ and *No* was coded as ‘no’. Maternal education was assessed at 32 weeks gestation using educational qualifications in common use at the time in the UK. Considering different school systems across countries, we coded this variable as ‘no high school’ (*CSE/none* or *vocational*), ‘high school’ (*O-level*), or ‘beyond high school’ (*A-level* or *degree*).

### Data analysis plan

We applied latent class growth analysis (LCGA) to identify developmental trajectories of conduct problems, using a bias-adjusted 3-step approach [42, 43]. This method accounts for misclassification error rates in latent class membership when estimating the effect of covariates [42, 43].

First, an *unconditional* latent class model was estimated (i.e., the meaning of classes was exclusively based on the SDQ conduct problems subscale, without being influenced by covariates). We addressed missing data in this model using a full information maximum likelihood estimator with robust standard errors (i.e., parameters were estimated using all available data). This missing data method has been shown to produce unbiased parameter estimates compared to listwise deletion, especially under the missing at random data loss mechanism and where there are higher rates of missing data [44]. We modeled linear, quadratic, and cubic patterns of change, each with between one and six class solutions. The following model fit indices were used to select the optimal class model: Bayesian Information Criterion (BIC) and sample size adjusted BIC (SSABIC), which are used to reduce the risk of overfitting the model to a single sample (lower values indicate a better model fit), and the Lo-Mendell-Rubin Likelihood Ratio Test (LMR-LRT), adjusted LMR-LRT, and Bootstrapped Likelihood Ratio Test (BLRT), which compare two adjacent class models (significant *p*-values indicate a better fit of the *k* class model compared to the *k*-1 class model). We further considered entropy values (0.40, 0.60, and 0.80 represent low, medium, and high class separation, respectively), sample size of the smallest class, and interpretability of each class trajectory [43].

Second, after the best-fitting model was identified, the class membership information (i.e., most likely class) of each participant and misclassification error rates of each latent class were retrieved.

Third, to preserve the class membership information of the unconditional latent class model (step 1), we used the misclassification error rates obtained in step 2 when examining associations between child abuse and conduct problems trajectory membership. We addressed missing data in this *conditional* model using inverse probability weighting (IPW). Complete-case analysis may produce biased estimates if excluded cases are systematically different from those which were included. IPW can minimize this bias by allocating sampling weights to complete cases and thereby restoring total sample estimates [45]. IPW has been recommended over other techniques for handling missing data (e.g., multiple imputation) when participants have missing data on entire assessment waves, as opposed to single items, which is especially common in longitudinal research [45] (see Supplementary Table 1 for information on how weights were derived). We used multinomial logistic regression to estimate the association between childhood-only, adolescence-only, and ‘persistent’ abuse and latent classes of conduct problems. Multinomial logistic regression estimates multinomial odds ratios (or relative risk ratios); however, we refer to effects as odds ratios (usually used for two exhaustive categories) throughout the results section for clarity. We primarily focused on the ‘any abuse’ category, but subsequently tested for associations between abuse subtypes and conduct problem trajectories. All analyses were adjusted for child sex, housing tenure, maternal severe depression, maternal smoking, and maternal education.

#### Missing data

The conduct problems trajectory model was based on 10,648 participants (77.2% of the total ALSPAC sample; 51.4% boys), with missing data addressed using full information maximum likelihood. Complete data for physical, psychological, and sexual abuse and all covariates was available for 3127 participants (29.4% of those included in the conduct problems trajectory model; 35.9% boys). Those with versus without missing data on child abuse and/or covariates showed higher rates of conduct problems across all time points, albeit with small effect sizes (*r*s ranging between 0.08–0.09, all *p*s < .001). Furthermore, participants with missing data were more likely to be male (OR 2.47) and more likely to be classified as early-onset persistent (OR 1.56) or childhood-limited (OR 1.24), and less likely to be classified in the low conduct problems trajectory (OR 0.78) than participants without missing data (all *p*s < .01; see Supplementary Table 2 for all pairwise comparisons). The sample sizes in adjusted analyses for any, physical, psychological, and sexual abuse were 3172, 3275, 3295, and 3279, respectively. See Supplementary Figure 2 for the retention flow chart across measures/analyses.

## Results

### Conduct problem trajectories

**Fig. 1:**
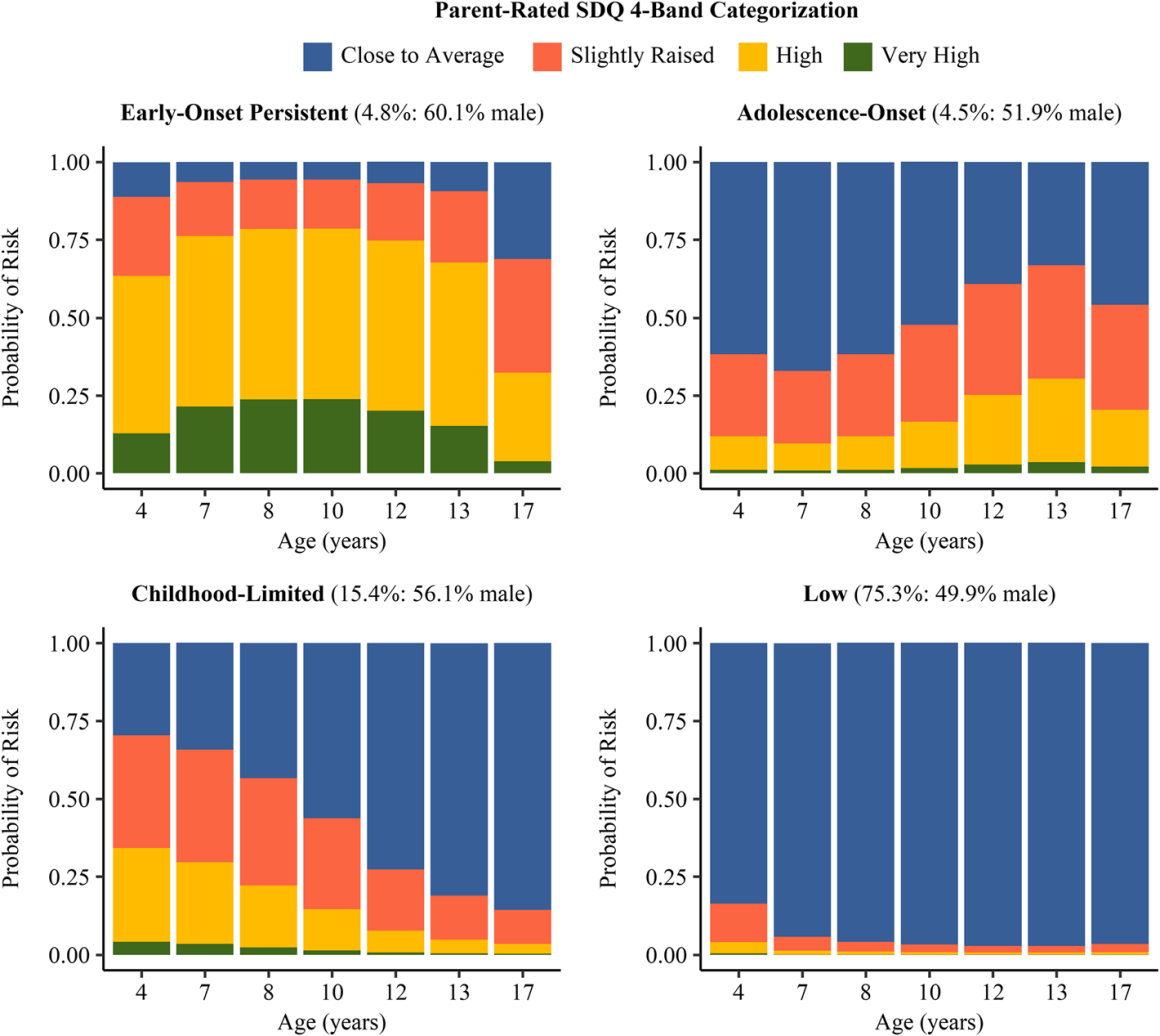
Predicted category proportions for each class in the conduct problems trajectory model (*N* = 10,648)

Models with cubic patterns of change yielded the best combination of model fit indices, interpretability of class trajectories, class sample sizes, and consistency with previous longitudinal research [46], including prior modeling of conduct problem trajectories in the ALSPAC sample [30]. Most of the model fit indices suggested that the 5- or 6-class models were the optimal models (e.g., lower BIC and SSABIC values, and statistically significant *p*-values for the LMR-LRT and BLRT). However, the results of the 5- and 6-class models were questionable because of two early-onset persistent class variants (low vs. high), with small sample sizes (< 2%). The existence of such classes at the population level is doubtful, as they have not been reported in previous longitudinal research [46], including prior latent growth modeling in the ALSPAC sample [30]. Additionally, such small class sizes are unlikely to be useful in subsequent analysis. The 4-class model (BIC = 57,164; SSABIC = 57,097) had better fit indices compared to the 3-class model (BIC = 57,311; SSABIC = 57,260) and each class was an acceptable size. We therefore rejected the 5- and 6-class models in favor of the 4-class model. The four classes and their respective proportions of the overall sample were: early-onset persistent (4.8%), adolescence-onset (4.5%), childhood-limited (15.4%), and low (75.3%) conduct problems (see Table 1 for model fit statistics). Figure 1 presents the plots of predicted SDQ category proportions of the 4-class model. In sum, the early-onset persistent class showed particularly high rates of ‘high’ conduct problems across all assessment waves, while the childhood-limited class showed a sharp and persistent decline in elevated conduct problems. The adolescence-onset class showed ‘slightly raised’ conduct problems in childhood and a continuous increase of ‘high’ conduct problems in adolescence. Finally, the low class showed predominantly ‘close to average’ conduct problems across all assessment waves.

**Table 1:** Model fit statistics for cubic latent class growth analysis one to six class solutions

Fit statistics	1 class	2 classes	3 classes	4 classes	5 classes	6 classes
LL (No. of Para.)	−32,500.791 (6)	−29,037.350 (11)	−28,581.588 (16)	− 28,484.751 (21)	−28,388.525 (26)	− 28,354.757 (31)
BIC	65,057.220	58,176.705	57,311.546	57,164.237	57,018.152	56,996.981
SSABIC	65,038.153	58,141.749	57,260.700	57,097.502	56,935.527	56,898.467
Entropy		0.763	0.725	0.709	0.688	0.697
LMR-LRT *p*-value		0.0000	0.0000	0.0403	0.0016	0.1998
Adj. LMR-LRT *p*-value		0.0000	0.0000	0.0426	0.0018	0.2061
BLRT *p*-value		0.0000	0.0000	0.0000	0.0000	0.0000
Group size (%)^a^						
C1	10,648	8598 (80.7%)	447 (4.2%)	480 (4.5%)	135 (1.3%)	1566 (14.7%)
C2		2050 (19.3%)	2343 (22.0%)	1643 (15.4%)	465 (4.3%)	7691 (72.2%)
C3			7858 (73.8%)	8019 (75.3%)	1616 (15.2%)	84 (0.8%)
C4				506 (4.8%)	7720 (72.5%)	167 (1.6%)
C5					712 (6.7%)	597 (5.6%)
C6						543 (5.1%)

***Note.*** Based on *N = *10,648, *LL* Log-Likelihood value, *No. of Para.* Number of estimated (freed) parameters, *BIC* Bayesian Information Criterion, *SSABIC* Sample Size Adjusted BIC, *LMR-LRT* Lo-Mendell-Rubin Likelihood Ratio Test, *Adj*. *LMR-LRT* Adjusted LMRT-LRT, *BLRT* Bootstrapped Likelihood Ratio Test, *C* Class. ^a^ Based on most likely latent class membership

#### Validation of derived conduct problem trajectories

Those with early-onset persistent, adolescence-onset, and childhood-limited conduct problems had significantly increased odds of showing antisocial behavior at age 14 years (2.6, 2.9, and 1.6 times greater odds, respectively) and 18 years (1.9, 1.9, and 1.6 times greater odds, respectively) compared to those with low conduct problems (all *p*s < .05), as measured using the ESYTC self-report measure. Additionally, at age 14 years, those with early-onset persistent and adolescence-onset conduct problems had 1.8 and 1.7 times greater odds, respectively, of showing antisocial behavior compared to those with childhood-limited conduct problems (all *p*s < .05; see Supplementary Table 3 for all pairwise comparisons).

### Descriptive statistics

Overall, across abuse exposure categories, 19.6% of the sample reported experiencing at least some form of abuse (i.e., ‘any abuse’), with 11.3, 8.9, and 8.1% of the sample reporting physical, psychological, and sexual abuse, respectively. 40.9% of participants in the early-onset persistent and 37.5% in the adolescence-onset conduct problem classes reported experiencing some form of abuse, compared with 23.8% of the childhood-limited and 16.8% of the low classes. For specific types of abuse, the proportions for the early-onset persistent, adolescence-onset, childhood-limited, and low conduct problem classes were: 31.0, 25.9, 13.1, and 9.3% for physical abuse; 28.5, 21.3, 11.1, and 6.9% for psychological abuse; and 11.7, 10.2, 10.7, and 7.3% for sexual abuse, respectively. Frequencies of exposure in each of the developmental phases (i.e., childhood-only, adolescence-only, and ‘persistent’ abuse) for the four conduct problem classes are presented in Table 2. Descriptive statistics of sociodemographic variables in the analysis sample can be found in Supplementary Table 1.

**Table 2:** Frequencies of abuse exposure in each of the developmental phases for the four conduct problem trajectories

	Low *n* (%)	CL *n* (%)	AO *n* (%)	EOP *n* (%)
**Any abuse** (*N* = 3172)				
Childhood-only	118 (4.7)	33 (7.7)	13 (9.0)	12 (10.9)
Adolescence-only	140 (5.6)	26 (6.1)	11 (7.6)	8 (7.3)
‘Persistent’	161 (6.5)	43 (10.0)	30 (20.8)	25 (22.7)
**Physical abuse** (*N* = 3275)				
Childhood-only	88 (3.4)	21 (4.7)	11 (7.5)	11 (9.5)
Adolescence-only	55 (2.1)	10 (2.2)	7 (4.8)	9 (7.8)
‘Persistent’	95 (3.7)	28 (6.2)	20 (13.6)	16 (13.8)
**Psychological abuse** (*N* = 3295)				
Childhood-only	50 (1.9)	18 (4.0)	7 (4.7)	10 (8.6)
Adolescence-only	48 (1.9)	12 (2.7)	8 (5.3)	7 (6.0)
‘Persistent’	80 (3.1)	20 (4.5)	17 (11.3)	16 (13.8)
**Sexual abuse** (*N* = 3279)				
Childhood-only	49 (1.9)	14 (3.1)	5 (3.4)	< 5
Adolescence-only	111 (4.3)	24 (5.4)	8 (5.4)	5 (4.5)
‘Persistent’	29 (1.1)	10 (2.2)	< 5	< 5

***Note.*** Sample sizes based on complete data. Cells with a count of < 5 were not included in subsequent analyses. *Childhood-only* Before age 11 years, *Adolescence-only* Between ages 11–17 years, *‘Persistent’* Before age 11 years AND between ages 11–17 years, *AO* Adolescence-onset, *CL* Childhood-limited, *EOP* Early-onset persistent

### Associations between child abuse and conduct problem trajectories

*Weighted* analyses are presented in Table 3, with all abuse comparisons being relative to those not exposed to any abuse. The strongest effects were observed for abuse that was reported in both childhood and adolescence. This ‘persistent’ abuse was associated with an 8- to 10-fold increase in the odds of being in the early-onset persistent and adolescence-onset classes compared to the low conduct problems class. In addition, ‘persistent’ abuse was associated with a 6- to 8-fold increased odds of being in the early-onset persistent and adolescence-onset classes compared to the childhood-limited conduct problems class. There was no evidence that exposure to ‘persistent’ abuse differentiated between the early-onset persistent and adolescence-onset conduct problem trajectories, or was associated with increased odds of being in the childhood-limited conduct problems class (versus the low class).

**Table 3:** Associations between developmental timing of abuse (collapsing across abuse subtypes to form an ‘any abuse’ category) and conduct problems trajectory membership

	Timing of abuse					
	Childhood-only		Adolescence-only		‘Persistent’	
	**OR**	**95% CI**	**OR**	**95% CI**	**OR**	**95% CI**
**Weighted**						
Low^a^						
CL	1.44	0.49–4.18	0.70	0.21–2.35	1.24	0.41–3.75
AO	**3.98**	**1.40–11.34**	2.72	0.97–7.57	**7.51**	**3.42–16.48**
EOP	**6.17**	**2.39–15.94**	2.89	0.81–10.32	**9.80**	**4.45–21.58**
CL^a^						
AO	2.77	0.48–15.98	3.87	0.62–24.28	**6.04**	**1.30–28.15**
EOP	4.29	0.97–18.97	4.12	0.64–26.60	**7.89**	**1.89–32.98**
AO^a^						
EOP	1.55	0.39–6.13	1.06	0.20–5.57	1.31	0.42–4.02
**Unweighted**						
Low^a^						
CL	1.65	0.76–3.60	0.92	0.34–2.44	0.98	0.33–2.97
AO	**3.24**	**1.27–8.26**	2.50	0.97–6.45	**6.48**	**3.31–12.68**
EOP	**3.94**	**1.71–9.02**	1.99	0.67–5.86	**6.75**	**3.49–13.06**
CL^a^						
AO	1.96	0.50–7.69	2.73	0.56–13.34	**6.59**	**1.53–28.34**
EOP	2.38	0.75–7.56	2.17	0.49–9.66	**6.87**	**1.77–26.72**
AO^a^						
EOP	1.21	0.35–4.17	0.79	0.18–3.49	1.04	0.42–2.61

***Note.*** Based on *N* = 3192. Inverse probability weighting was used to allocate sampling weights to complete cases in weighted analysis. In unweighted analysis, each case carries the same weight. All pairwise comparisons are adjusted for child sex, housing tenure, maternal severe depression, maternal smoking, and maternal educational level. Bold values indicate statistically significant associations. ^a^ Reference group. Key: *Childhood-only* Before age 11 years, *Adolescence-only* Between ages 11–17 years, *‘Persistent’* Before age 11 years AND between ages 11–17 years, *AO* Adolescence-onset, *CL* Childhood-limited, *EOP* Early-onset persistent, *OR* Multinomial odds ratio

The effects for childhood-only abuse were similar, albeit slightly weaker. Childhood-only versus no abuse was associated with a 4- to 6-fold increased odds of being in the early-onset persistent and adolescence-onset classes relative to the low conduct problems class. However, it did not distinguish these classes from each other or from the childhood-limited conduct problems class, nor was it associated with increased odds of being in the childhood-limited compared to the low conduct problems class. Lastly, we found no evidence that adolescence-only abuse was associated with conduct problems trajectory membership – which may partly reflect the fact that adolescence-only abuse was rarer than childhood-only or ‘persistent’ abuse.

In sum, we found relatively robust associations between abuse occurring either in childhood alone or in both childhood and adolescence and the early-onset persistent and adolescence-onset conduct problem trajectories. We found no evidence that abuse occurring in either childhood and/or adolescence was associated with childhood-limited conduct problems, and abuse occurring only in adolescence was not associated with any elevated conduct problems trajectory. *Unweighted* analyses, which showed the same pattern of associations albeit with slightly weaker effects, are also provided in Table 3 for comparison purposes.

### Exploratory analyses assessing abuse subtypes

*Weighted* analyses are presented in Table 4 (*unweighted* results were similar and are available on request), with those not exposed to the respective abuse subtype serving as the reference group in each case. In contrast to the pattern of effects observed for the any abuse category, physical and psychological abuse showed strong effects across all three developmental periods studied. More precisely, childhood-only, adolescence-only, and ‘persistent’ physical abuse was associated with a 4- to 8-fold increase in the odds of being in the early-onset persistent and adolescence-onset classes compared to the low conduct problems class. Similarly, childhood-only, adolescence-only, and ‘persistent’ psychological abuse was associated with a 5- to 11-fold increase in the odds of being in the early-onset persistent and adolescence-onset classes versus the low conduct problems class (although the association between childhood-only psychological abuse and adolescence-onset trajectory membership was not significant). Similar to the findings for any abuse, there was no evidence that exposure to physical or psychological abuse across childhood and/or adolescence differentiated between the early-onset persistent and adolescence-onset conduct problem trajectories, or was associated with the childhood-limited conduct problem trajectory. For sexual abuse, the early-onset persistent and adolescence-onset classes showed cell counts of less than 5 for some developmental periods. Therefore, meaningful analyses of associations between sexual abuse and conduct problem classes could not be performed.

**Table 4:** *Weighted* associations between physical (versus no physical) and psychological (versus no psychological) abuse and conduct problems trajectory membership

	Timing of abuse subtypes					
	Childhood-only		Adolescence-only		‘Persistent’	
	**OR**	**95% CI**	**OR**	**95% CI**	**OR**	**95% CI**
**Physical abuse** (*N* = 3275)						
Low^a^						
CL	0.93	0.21–4.14	N/A	N/A	1.73	0.57–5.20
AO	**4.50**	**1.63–12.43**	**3.86**	**1.43–10.38**	**7.76**	**3.20–18.82**
EOP	**4.99**	**1.91–13.03**	**6.00**	**2.29–15.74**	**7.60**	**3.24–17.82**
CL^a^						
AO	4.85	0.63–37.24	N/A	N/A	4.49	0.97–20.73
EOP	5.37	0.93–31.10	N/A	N/A	**4.39**	**1.04–18.59**
AO^a^						
EOP	1.11	0.29–4.27	1.56	0.37–6.46	0.98	0.30–3.17
**Psychological abuse** (*N* = 3295)						
Low^a^						
CL	1.52	0.28–8.22	1.58	0.32–7.79	0.80	0.11–5.66
AO	4.16	0.87–19.85	**4.96**	**1.59–15.45**	**5.48**	**2.43–12.33**
EOP	**10.48**	**4.04–27.15**	**10.83**	**3.45–34.01**	**11.22**	**4.97–25.36**
CL^a^						
AO	2.74	0.18–42.32	3.14	0.39–25.09	6.84	0.72–64.79
EOP	**6.90**	**1.06–44.78**	6.86	0.83–56.99	N/A	N/A
AO^a^						
EOP	2.52	0.44–14.29	2.18	0.47–10.15	2.05	0.70–6.02

***Note.*** Inverse probability weighting was used to allocate sampling weights to complete cases. N/A = Not available due to fixed parameters. All pairwise comparisons are adjusted for child sex, housing tenure, maternal severe depression, maternal smoking, and maternal educational level. Bold values indicate statistically significant associations. ^a^ Reference group. *Key: Childhood-only* Before age 11 years, *Adolescence-only* Between ages 11–17 years, *‘Persistent’* Before age 11 years AND between ages 11–17 years, *AO* Adolescence-onset, *CL* Childhood-limited, *EOP* Early-onset persistent, *OR* Multinomial odds ratio

## Discussion

Using data from a prospective longitudinal study with a large, population-based sample, we identified developmental trajectories of conduct problems from ages 4–17 years, and investigated links between abuse experienced at different times during development and the derived conduct problem trajectories. In contrast to previous research using developmental trajectories of conduct problems that focused on abuse experienced during childhood [17–20], we used measures covering both childhood and adolescence, which enabled us to explore the impact of abuse timing and persistence. We found that abuse exposure was associated with substantially greater odds of being in the early-onset persistent and adolescence-onset conduct problem classes, particularly when it was present across both childhood and adolescence. We did not find stronger associations between child abuse and membership of the early-onset persistent compared to the adolescence-onset class, which is in contrast to some previous findings [17–19]. However, it has to be noted that the adolescence-onset class showed slightly raised conduct problems already in childhood, a pattern that has also been observed in prior modeling of conduct problem trajectories [19, 20, 30]. We also did not replicate previous findings showing an association between abuse exposure and increased odds of being in the childhood-limited class (compared to the low conduct problems class) [19, 20]. Overall, our findings suggest that conduct problems with an onset in adolescence show similar associations with abuse to conduct problems that emerge in childhood and persist, with any differences between these trajectories being quantitative (i.e., implying common risk factors) rather than qualitative (i.e., distinct risk factors) in nature.

We extended previously published conduct problem trajectories from ages 4–13 years up to age 17 years in a large UK birth cohort [30]. Using a full information maximum likelihood estimator and the updated 4-band categorization of the SDQ conduct problems subscale, we were able to increase the sample size (*N* = 10,648) and capture more variability in conduct problems, compared to the sample size previously used to estimate developmental trajectories (*N* = 7218), which also used a dichotomous approach, classifying individuals as either ‘high risk’ or ‘not high risk’ in terms of conduct problems [30]. This has the potential to enable other researchers to examine associations between other environmental or genetic risk factors and conduct problem trajectories covering both childhood and adolescence. Furthermore, the current study brings together two areas of developmental psychopathology, namely: (i) studies using conduct problem trajectories, which, however, measured child abuse exclusively during childhood, rather than in adolescence or in both developmental periods [17–20]; and (ii) studies examining the impact of timing of child abuse, which have been limited to adolescent and adult antisocial behavior, rather than developmental trajectories [26–29].

In line with official UK government statistics from 2020 on child abuse in England and Wales [47], we found that one in five participants (19.6%) reported at least one form of child abuse (i.e., ‘any abuse’). Prevalence rates for specific types of abuse were also broadly comparable with official statistics, ranging from 8 to 11%. The current study was limited to child abuse, rather than child neglect. Thus, comparisons with official statistics on the prevalence of neglect are not possible.

The current study builds on previous research by examining timing of exposure to child abuse in relation to developmental trajectories of conduct problems. Importantly, our findings support the hypothesis that persistent abuse has a more detrimental effect than time-limited abuse [25]. Thus, in line with the cumulative risk hypothesis, abuse exposure in both childhood and adolescence was associated with greater odds of being in the early-onset persistent and adolescence-onset classes, with effect sizes twice the size of those observed for childhood-only abuse. In addition, different patterns were observed for childhood-only versus adolescence-only exposure when using the aggregate measure of abuse (‘any abuse’). Specifically, whereas childhood-only abuse was associated with increased odds of being in the early-onset persistent and adolescence-onset conduct problem classes, adolescence-only abuse was not associated with membership of any of the elevated conduct problem trajectories. The latter observation runs counter to previous research suggesting that adolescence-only abuse has more detrimental effects than childhood-only abuse [26–28]. On the contrary, the current results indicate that abuse occurring in childhood may be more influential than that occurring in adolescence (at least in terms of increasing risk for conduct problems), suggesting there may be a sensitive period in which abuse is particularly likely to lead to persistent conduct problems. Alternatively, it may be that abuse occurring specifically in adolescence, versus in childhood or in both developmental periods, is experienced differently by the individual or arises for different reasons, given that significant conflict in the parent-child relationship is relatively common (and possibly normative) during adolescence [48].

These findings for childhood-only versus adolescence-only abuse were not replicated in an exploratory analysis that examined the impacts of physical and psychological abuse separately. More precisely, adolescence-only abuse also emerged as predictive of these trajectories, alongside the positive associations already identified for childhood-only abuse. In line with many studies published in this field, small cell sizes mean that caution is essential in interpreting these findings. They also prevented us from investigating the specific impact of sexual abuse, which has been consistently linked to adolescent conduct problems [15, 16], because of particularly low frequencies in our sample when split across conduct problem classes. As shown in the correlation matrix (see Supplementary Fig. 1), physical and psychological abuse were highly correlated, whereas correlations between these forms of abuse and sexual abuse were much weaker. This may indicate shared risk environments in which both physical and psychological abuse occur, which may explain the similar pattern of effects for these abuse subtypes. Although sexual abuse was most commonly reported in adolescence, it might be less likely to result in conduct problems if experienced within this developmental period. Consequently, the inclusion of sexual abuse in our aggregate measure of abuse may have suppressed associations with adolescence-only abuse. Future studies with larger and/or high-risk samples with a higher prevalence of sexual abuse are needed to further investigate the association between sexual abuse and conduct problem trajectories.

In contrast to the effects observed for the early-onset persistent and adolescence-onset classes, we did not find any evidence of associations between abuse and childhood-limited conduct problems, which contradicts some previous findings in this area [19, 20]. These studies, however, also included child neglect, a form of child maltreatment not investigated in the current study, which may have influenced associations. Alternatively, individual risk factors, such as neurodevelopmental problems, may be particularly pronounced in these individuals [11], and, thus, more relevant in the etiology of this trajectory compared to environmental risk factors such as child abuse. For example, Raine et al. (2005) found a range of neurocognitive impairments related to intelligence and memory especially in children with childhood-limited conduct problems compared to those on the low trajectory [19].

The relationship between child abuse and the early-onset persistent and adolescence-onset conduct problem trajectories may be explained with recourse to social information processing theory [49]. Children with aggressive behavior show biases in social information processing (e.g., hostile attributional biases) [50]. These biases have been shown to mediate the relationship between harsh and abusive parenting and conduct problems [51, 52]. Children may internalize their parents’ aggressive and threatening behaviors, and, as a result, rely on these aggressive schemata in future social interactions. Equipped with this limited repertoire of behaviors, children may struggle to generate non-aggressive responses to situations of conflict and may also evaluate physically and verbally aggressive responses more positively than their non-abused peers [49]. Furthermore, there are well-established bidirectional effects in the relationship between harsh and abusive parenting and child conduct problems [53]. Consequently, children showing conduct problems may become ensnared in coercive exchanges with their parents [54]. By contrast, abuse experienced in adolescence might be less likely to be internalized and viewed as a behavior to emulate, which may explain the null findings for adolescence-only abuse when using the aggregate measure of abuse. Alternatively, adolescence-only abuse may be more relevant for other types of antisocial behavior, which are not assessed by the SDQ conduct problems subscale, and other forms of psychopathology. For example, Mersky et al. (2012) found that adolescence-only abuse was linked to juvenile offending (i.e., arrests, court petitions, and various types of offenses) and particularly drug-related convictions in adulthood [29]. Finally, the non-significant associations for adolescence-only abuse may partly reflect the relative rarity of abuse only occurring in this developmental period.

### Limitations

First, the findings should be interpreted in the context of limitations relating to our measures of abuse in the current study. A highly varied set of experiences could lead to an individual being classified as having experienced child abuse. This problem is inherent in any measure that attempts to capture something as complex as exposure to adversity in a scale score, but is compounded in cohort studies where low prevalence of child abuse necessitates the use of categorical variables. In addition, child abuse was assessed using retrospective self-report at age 22 years, which may have been subject to recall bias. Prospective and retrospective measures of child abuse often show poor agreement, representing two constructs with limited overlap [55]. However, despite this discrepancy, false positives of retrospective reports of child abuse in adulthood have been shown to be rare [56]. Furthermore, most instances of child abuse are not reported to authorities [57], which compromises the representativeness of officially documented child abuse cases – the main alternative to self-report. Therefore, while retrospective self-report measures have limitations, it is difficult to develop feasible and ethically acceptable alternatives, particularly in large prospective cohort studies. We further used a brief measure of child abuse which has not been fully validated, although the included items are extremely similar to those included in well-established measures (e.g., Childhood Trauma Questionnaire [58]). Future studies need to replicate our findings using a larger number of items from a measure with established psychometric properties. Finally, our measure of ‘persistent’ abuse, defined as exposure to abuse occurring in both childhood and adolescence, may have captured two isolated instances of abuse, rather than a repeated and ongoing pattern of abuse that spans childhood and adolescence. Unfortunately, the available data did not permit a more detailed approach but this issue merits investigation in future research. Second, as already noted, despite this study deriving from a large, representative birth cohort, frequencies of some forms of abuse were low across the different conduct problem trajectories. The findings relating to physical and psychological abuse particularly require replication, and we were not able to examine sexual abuse as a separate category due to very small cell counts for some classes. Third, the present study suffered from high attrition rates, possibly due to the high assessment burden and/or participants’ unwillingness to answer questions about highly intrusive experiences, potentially resulting in systematic differences between the sample included in the analyses estimating conduct problem trajectories (*N* = 10,648) and the sample for whom retrospective data on child abuse were available (*n* = 3172). More precisely, those with missing data were more likely to be male and in the early-onset persistent and childhood-limited conduct problem classes. This may have led to an underestimation of the effects of abuse and compromised the generalizability of our findings, particularly given that conduct problem trajectories where associated with missingness. However, we employed IPW to minimize the impact of this bias by allocating sample weights to complete cases [45], and the findings of weighted and unweighted analyses yielded almost identical results. Fourth, the SDQ conduct problems subscale showed modest internal consistency, similar to previous research modeling developmental trajectories of behavior and emotional problems in the ALSPAC sample [59]. Although, the SDQ is an extremely widely used measure, our findings require replication, using a measure of conduct problems with better psychometric properties. Similarly, the ESYTC, which we used to validate the derived conduct problem trajectories, showed poor reliability. Collectively, these limitations highlight the need for more reliable measures of behavior problems in young people. Fifth, relying on parent-reported conduct problems in adolescence may have underestimated the level of behavioral problems, as parents may be unaware of their child’s antisocial behavior in this developmental phase [60]. However, the use of different informants for conduct problems versus abuse experiences minimizes potential for inflation of effects by informant bias. Moreover, considering the age range of our sample from ages 4–17 years, neither parent- nor self-report would have perfectly captured conduct problems occurring in both childhood and adolescence. Crucially, we were able to validate our conduct problem trajectories using self-reported measures of antisocial behavior during adolescence, which showed higher rates of antisocial behavior in the elevated conduct problems classes. This information, which is not typically available for studies of this type, supports the validity of our derived trajectories. Nevertheless, an important area of future research will be to compare associations between child abuse and developmental trajectories of conduct problems based on self- versus parent-report. Finally, the temporal overlap between our derived conduct problem trajectories and measures of child abuse precludes causal inferences. Thus, child abuse may be a risk factor for conduct problems or conduct problems may elicit more harsh and abusive parenting, or both factors may interact with each other in a transactional way.

## Conclusions

Our findings demonstrate a particularly strong association between ‘persistent’ abuse – i.e., that occurring in both childhood and adolescence – and the early-onset persistent and adolescence-onset conduct problem trajectories. The findings are consistent with the view that the differences between the early-onset persistent and adolescence-onset conduct problem trajectories are more quantitative than qualitative in nature. In other words, common risk factors are involved in both subtypes but to different degrees, rather than early-onset persistent conduct problems stemming from entirely different risk factors compared to adolescence-onset conduct problems. For example, levels of exposure to environmental risk factors, such as child abuse, may be more similar than previously thought, as direct comparisons between these conduct problems trajectories revealed no significant differences in abuse exposure. Consequently, child services may want to screen for a history of child abuse and provide additional support to young people showing adolescence-onset conduct problems, as these may not be as developmentally normative as previously suggested [9]. Thus, psychosocial interventions focusing on ameliorating adverse family environments may be also effective in reducing adolescence-onset conduct problems. Furthermore, studies of the effectiveness of interventions aimed at targeting harsh and abusive parenting should assess outcomes in adolescence, as well as outcomes that are concurrent with the delivery of the intervention – as the full benefits may not be apparent until many years later. Our findings also demonstrate the importance of adopting measures covering both childhood and adolescence when investigating the timing and persistence of child abuse, as harsh and abusive parenting may persist up to emerging adulthood.

## Supplementary Information

**Additional file 1: Supplementary Figure 1.** Tetrachoric correlation matrix of physical, psychological, and sexual abuse occurring in either childhood or adolescence. **Supplementary Table 1.** Associations between indicators used to derive the inverse probability weights and inclusion in the analysis sample of ‘any abuse’. **Supplementary Table 2.** Descriptive statistics and group comparisons between participants with class membership information (*N* = 10,648) and with and without complete data on measures of child abuse and covariates (*N* = 3127). **Supplementary Figure 2.** Retention flow chart across measures/analyses. **Supplementary Table 3.** Validation of conduct problem classes against an independent measure of self-reported antisocial behavior

## Data Availability

The study website contains details of all the data that are available through a fully searchable data dictionary and variable search tool (http://www.bristol.ac.uk/alspac/researchers/our-data/).

## Abbreviations

ALSPAC: Avon Longitudinal Study of Parents and Children
BIC: Bayesian Information Criterion
BLRT: Bootstrapped Likelihood Ratio Test
ESYTC: Edinburgh Study of Youth Transitions and Crime
IPW: Inverse probability weighting
LCGA: Latent class growth analysis
LMR-LRT: Lo-Mendell-Rubin Likelihood Ratio Test
SDQ: Strengths and Difficulties Questionnaire
SSABIC: Sample size adjusted Bayesian Information Criterion
