# Supplementary material for: Associations between developmental timing of child abuse and conduct problem trajectories in a UK birth cohort
Source: BMC Psychiatry. 2021 Mar 16;21:89. doi: 10.1186/s12888-021-03083-8 (PMC7962332; doi:10.1186/s12888-021-03083-8)
Supplement: Supplementary file 1 — Additional file 1: Supplementary Figure 1. Tetrachoric correlation matrix of physical, psychological, and sexual abuse occurring in either childhood or adolescence. Supplementary Table 1. Associations between indicators used to derive the inverse probability weights and inclusion in the analysis sample of ‘any abuse’. Supplementary Table 2. Descriptive statistics and group comparisons between participants with class membership information (N = 10,648) and with and without complete data on measures of child abuse and covariates (N = 3127). Supplementary Figure 2. Retention flow chart across measures/analyses. Supplementary Table 3. Validation of conduct problem classes against an independent measure of self-reported antisocial behavior [file 12888_2021_3083_MOESM1_ESM.docx]

**Supplementary Figure 1** Tetrachoric correlation matrix of physical, psychological, and sexual abuse occurring in either childhood or adolescence


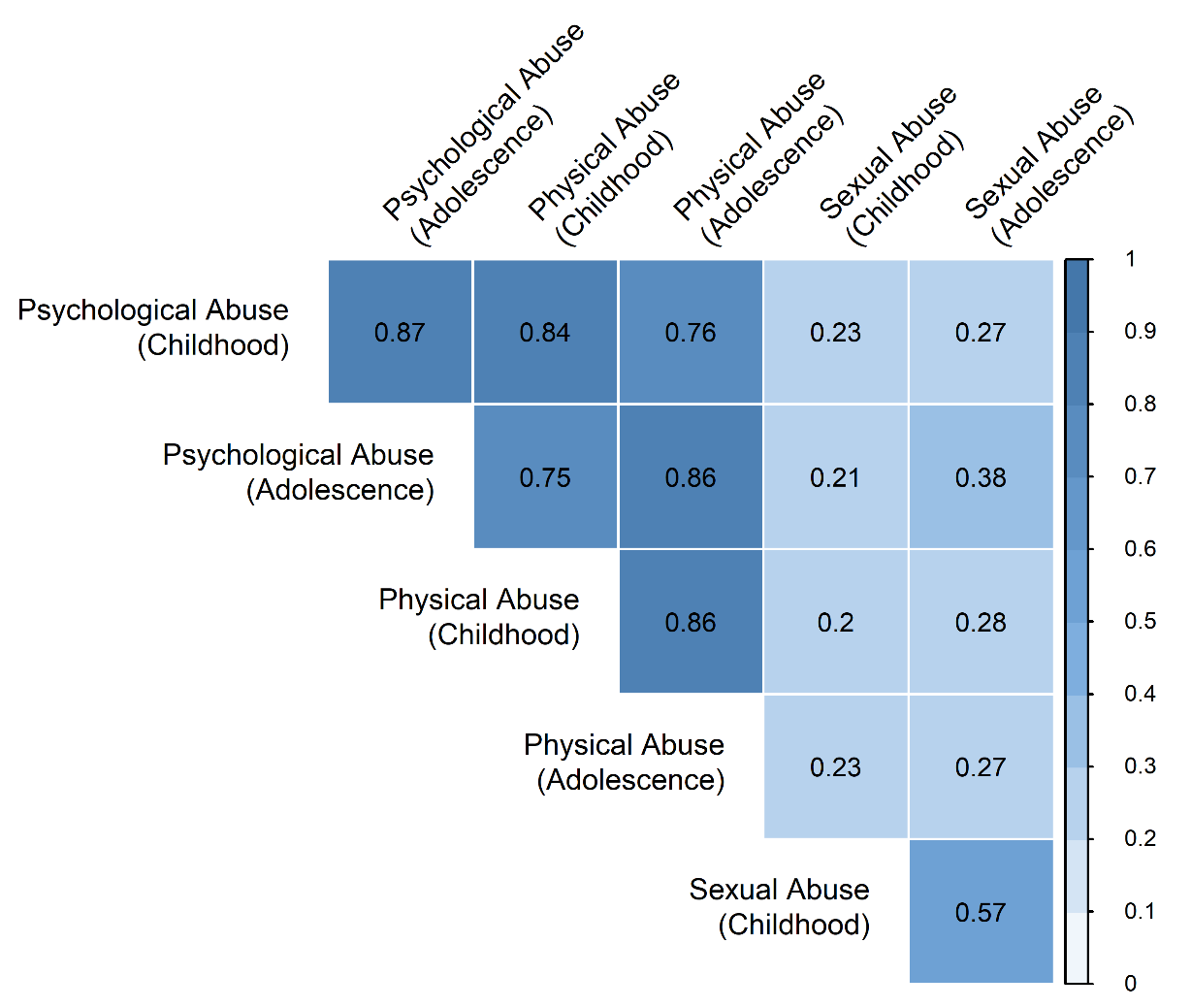


***Note.*** Based on *N* = 3497. All *p*s < .001. Darker colors represent larger correlation coefficients.

| **Supplementary Table 1** Associations between indicators used to derive the inverse probability weights and inclusion in the analysis sample of ‘any abuse’ | | | | | | |
| --- | --- | --- | --- | --- | --- | --- |
| **Sociodemographic and parental factors in pregnancy** | | **In analysis sample** % (*n*) | | **Not in analysis sample**  % (*n*) | **OR (95% CI)** | ***p*-value** |
|  | | | (*N* = 3172) | (*N* = 7476) |  |  |
| **Child sex** | | |  |  |  |  |
|  | Female | | 64.1% (2034) | 42.0% (3140) | 0.41 (0.37-0.44) | < .001 |
| **Maternal education** | | |  |  |  |  |
|  | No high school | | 16.5% (522) | 28.7% (2143) | Reference |  |
|  | High school | | 33.7% (1070) | 34.2% (2556) | 0.58 (0.52-0.66) | < .001 |
|  | Beyond high school | | 49.8% (1580) | 37.1% (2777) | 0.43 (0.38-0.48) |  |
| **Maternal smoking** | | |  |  |  |  |
|  | Yes | | 15.1% (479) | 24.2% (1807) | 1.79 (1.60-2.01) | < .001 |
| **Housing tenure** | | |  |  |  |  |
|  | Other^a^ | | 13.8% (438) | 23.9% (1787) | 1.96 (1.75-2.20) | < .001 |
| ***Note.*** ^a^ Reference is ‘mortgaged/owned’. Outcome coded as 0 ‘in analysis sample’ and 1 ‘not in analysis sample’. | | | | | | |

Measures assessed in pregnancy (child sex, maternal education, maternal smoking, and housing tenure) that were independently predictive of child abuse exposure, conduct problem trajectories, or missingness in the analysis sample were selected as inverse probability weighting (IPW) indicators. Due to slightly varying attrition rates across child abuse variables, we looked at missingness in each group separately, resulting in four missingness models (i.e., different weights for any, physical, psychological, and sexual abuse). Missing data on IPW indicators were singly imputed as the mode value (all indicators had < 7% of values missing, except maternal education with 11.1% of values missing). The Hosmer-Lemeshow test was used to assess model fit of the missingness models, with results showing no indication of poor fit (*p*-values ranging between .19 and .36). Weights ranged from 1.9 to 13.3. Due to almost identical values across missingness models, only associations between IPW indicators and inclusion in the analysis sample for ‘any abuse’ are shown in Table 1.

| **Supplementary Table 2** Descriptive statistics and group comparisons between participants with class membership information (*N* = 10648) and with and without complete data on measures of child abuse and covariates (*N* = 3172) | | | | | | | | |
| --- | --- | --- | --- | --- | --- | --- | --- | --- |
|  | | **In analysis sample** (*N* = 3172) | | **Not in analysis sample** (*N* = 7476) | | **Group comparison** | | **Effect size** |
|  | | **Mean (SD)** or **%** | ***N*** | **Mean (SD)** or **%** | ***N*** | ***t*(df)** or ***χ^2^*(df)** | ***r*** or **(95% CI)** | |
| **Conduct problems**^a^ | |  |  |  |  |  |  | |
|  | Age 4 | 0.37 (0.27) | 2919 | 0.40 (0.29) | 6355 | *t*(5920.2) = 5.91, *p* < .001 | 0.08 | |
|  | Age 7 | 0.29 (0.28) | 2802 | 0.33 (0.29) | 5287 | *t*(6020.9) = 6.33, *p* < .001 | 0.08 | |
|  | Age 8 | 0.27 (0.27) | 2721 | 0.31 (0.30) | 4637 | *t*(6026.5) = 6.53, *p* < .001 | 0.08 | |
|  | Age 10 | 0.22 (0.26) | 2701 | 0.26 (0.28) | 4531 | *t*(6051.1) = 6.04, *p* < .001 | 0.08 | |
|  | Age 12 | 0.20 (0.25) | 2631 | 0.25 (0.29) | 3996 | *t*(6125.7) = 7.27, *p* < .001 | 0.09 | |
|  | Age 13 | 0.21 (0.25) | 2608 | 0.26 (0.28) | 3799 | *t*(6006.2) = 6.69, *p* < .001 | 0.09 | |
|  | Age 17 | 0.16 (0.23) | 2378 | 0.21 (0.26) | 2687 | *t*(5061.9) = 6.54, *p* < .001 | 0.09 | |
| **Class proportions**^b^ | |  |  |  |  |  |  | |
|  | EOP | 3.5 | 110 | 5.3 | 396 | *χ^2^*(1) = 16.46, *p* < .001 | 1.56 (1.25 – 1.95) | |
|  | AO | 4.5 | 144 | 4.5 | 336 | *χ*^2^(1) = 0.01, *p* = .92 | 0.99 (0.81 – 1.22) | |
|  | CL | 13.5 | 428 | 16.3 | 1215 | *χ^2^*(1) = 12.99, *p* < .01 | 1.24 (1.10 – 1.40) | |
|  | Low | 78.5 | 2490 | 74.0 | 5529 | *χ^2^*(1) = 24.72, *p* < .001 | 0.78 (0.70 – 0.86) | |
| **Male** | | 35.9 | 1138 | 58.0 | 4336 | *χ^2^*(1) = 436.32, *p* < .001 | 2.47 (2.26 – 2.69) | |
| ***Note.*** ^a^ Assessed with the Strengths and Difficulties Questionnaire conduct problems subscale. ^b^ Based on most likely latent class membership. SD = Standard deviation; df = Degrees of freedom; *r* = Correlation coefficient; OR = Odds ratio; CI = Confidence interval; EOP = Early-onset persistent; AO = Adolescence-onset; CL = Childhood-limited. | | | | | | | | |

**Supplementary Figure 2** Retention flow chart across measures/analyses


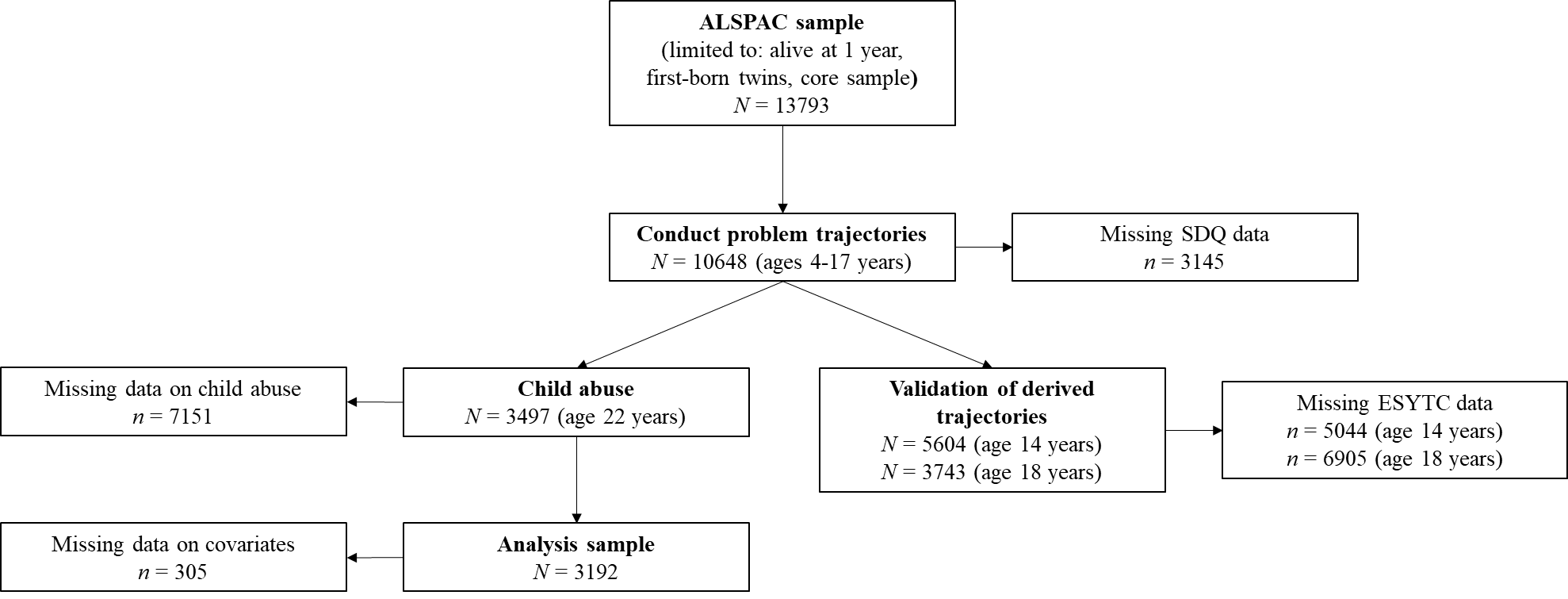


***Note.*** SDQ = Strengths and Difficulties Questionnaire; ESYTC = Edinburgh Study of Youth Transition and Crime.

| **Supplementary Table 3** Validation of conduct problem classes against an independent measure of self-reported antisocial behavior | | | | | |
| --- | --- | --- | --- | --- | --- |
|  | | **ESYTC** | | | |
|  | | **Age 14** (*N* = 5604) | | **Age 18** (*N* = 3743) | |
|  | | **OR** | **95% CI** | **OR** | **95% CI** |
| Low^a^ | |  |  |  |  |
|  | CL | **1.55** | **1.19-2.01** | **1.58** | **1.03-2.44** |
|  | AO | **2.85** | **1.87-4.33** | **1.85** | **1.00-3.42** |
|  | EOP | **2.56** | **1.77-3.69** | **1.89** | **1.11-3.21** |
| CL^a^ | |  |  |  |  |
|  | AO | **1.65** | **1.03-2.66** | 1.17 | 0.51-2.67 |
|  | EOP | **1.84** | **1.07-3.16** | 1.19 | 0.59-2.43 |
| ***Note.*** Bold values indicate statistically significant associations. ^a^ Reference group. Key: ESYTC = Edinburgh Study of Youth Transitions and Crime; AO = Adolescence-onset; CI = Confidence interval; CL = Childhood-limited; EOP = Early-onset persistent; OR = Multinomial odds ratio. | | | | | |
